# Recent insights into *Candida albicans* biofilm resistance mechanisms

**DOI:** 10.1007/s00294-013-0400-3

**Published:** 2013-08-25

**Authors:** Lotte Mathé, Patrick Van Dijck

**Affiliations:** 1 Department of Molecular Microbiology, VIB, Leuven, Belgium; 2 Laboratory of Molecular Cell Biology, KU Leuven, Kasteelpark Arenberg 31 bus 2438, Heverlee, 3001 Leuven, Belgium

**Keywords:** *Candida albicans*, Biofilm, Drug resistance, Host immune response

## Abstract

Like other microorganisms, free-living *Candida albicans* is mainly present in a three-dimensional multicellular structure, which is called a biofilm, rather than in a planktonic form. *Candida albicans* biofilms can be isolated from both abiotic and biotic surfaces at various locations within the host. As the number of abiotic implants, mainly bloodstream and urinary catheters, has been increasing, the number of biofilm-associated bloodstream or urogenital tract infections is also strongly increasing resulting in a raise in mortality. Cells within a biofilm structure show a reduced susceptibility to specific commonly used antifungals and, in addition, it has recently been shown that such cells are less sensitive to killing by components of our immune system. In this review, we summarize the most important insights in the mechanisms underlying biofilm-associated antifungal drug resistance and immune evasion strategies, focusing on the most recent advances in this area of research.

## Introduction

Being a commensal, *Candida albicans* is expected to inhabit the urogenital and gastrointestinal tract of a large percentage of the human population. In healthy individuals, its growth is confined by actions of the immune system and by the presence of other commensal microorganisms occupying its potential niche. However, when one of these barriers is disrupted, *C. albicans* can behave as a pathogen causing both superficial and systemic infections, the latter with possible infections of internal organs (Berman and Sudbery 2002; Kim and Sudbery 2011). Bloodstream infections are associated with considerable attributable mortality rates varying from 30 to 70 % (Bouza et al. 2013; Falagas et al. 2006; Kibbler et al. 2003; Peng and Lu 2013; Wey et al. 1988; Wisplinghoff et al. 2004) and high health-care costs with estimates ranging from millions to 1 billion dollars in the US alone (Miller et al. 2001; Wilson et al. 2002). Major risk factors for candidemia are neutrophil depletion and gastrointestinal damage, resulting in dispersion of *Candida* cells resident in the gastrointestinal tract to the bloodstream (Koh et al. 2008) and the frequent use of catheters in hospitalized patients that can present a substrate for the formation of biofilms (Darouiche 2004; Kim and Sudbery 2011; Kojic and Darouiche 2004). The risk of biofilm development on catheters has been estimated to be up to 30 %, depending on the location of the catheter (Ramage et al. 2006).

## Biofilm lifestyle of *Candida albicans*

Biofilms are defined as structured microbial communities that are attached to a surface and surrounded by a self-produced extracellular matrix (Costerton et al. 1995). In the early years, major focus was on bacterial biofilms, with a first model to study *C. albicans* biofilm development in vitro only emerging in 1994 (Hawser and Douglas 1994). Since then, ample model systems for the study of fungal biofilms have been developed (Tournu and Van Dijck 2012) and *C. albicans* biofilm formation has been characterized both in vitro and in vivo by several research groups (Andes et al. 2004; Chandra et al. 2001a, 2011; Řičicová et al. 2010). In general, *C. albicans* biofilm formation is characterized by four stages: (1) cell-wall protein-mediated adherence of yeast cells to a surface, (2) growth of the attached yeast cells into a thin layer of cells, (3) maturation of the biofilm through development of pseudohyphae and hyphae and excretion of matrix material and (4) dispersal of yeast cells from the biofilm possibly leading to colonization of distant places (Blankenship and Mitchell 2006; Chandra et al. 2001a; Kaneko et al. 2013; Uppuluri et al. 2010). Although biofilm structures can differ depending on the growth conditions (Baillie and Douglas 1998, 2000) mature *C. albicans* biofilms, mostly present after 24–48 h of biofilm formation (Andes et al. 2004; Kaneko et al. 2013; Řičicová et al. 2010), consist of a thin yeast layer responsible for attachment of the thicker layer, comprising both yeast and hyphal cells, to the surface (Baillie and Douglas 1999b). Structurally, several microcolonies can be distinguished which are separated by water channels allowing circulation of nutrients (Douglas 2003; Watnick and Kolter 2000). Over the past years, the genetic network controlling biofilm formation has been investigated and partially elucidated, both in vitro and in vivo (Banerjee et al. 2013; Bonhomme et al. 2011; Fanning et al. 2012; García-Sánchez et al. 2004; Murillo et al. 2005; Nett et al. 2009; Nobile et al. 2012). Discussion of the genetic control of biofilm formation is not the purpose of this review, but has been discoursed elsewhere (Finkel and Mitchell 2011; Fox and Nobile 2012; Nobile and Mitchell 2006).

Although *C. albicans* is still considered the most prevalent pathogen within the *Candida* clade, non- *albicans Candida* species are increasingly being isolated from patients, with *C. glabrata*, *C. parapsilosis* and *C. tropicalis* being the most represented ones (Horn et al. 2009; Peng and Lu 2013; Pfaller and Diekema 2007, 2010). Like *C. albicans*, these species are capable of forming biofilms (Hawser and Douglas 1994; Shin et al. 2002; Silva et al. 2010), be it to a lesser extent, increasing their potential to cause disease in patients with medical implant devices. Next to single species bloodstream infections associated with biofilms on medical implant devices, multi-species candidemia is also encountered, making up 4–8 % of all *Candida*-associated bloodstream infections (Klotz et al. 2007; Nace et al. 2009). Seemingly more prevalent with 7–27 % of all candidemias are polymicrobial bloodstream infections, in which *Candida* spp. are present together with bacteria such as *Enterococcus* spp., *Streptococcus* spp., *Staphylococcus aureus* and *Pseudomonas aeruginosa* (Bouza et al. 2013; Harriott and Noverr 2010; Klotz et al. 2007).

Biofilms are often regarded as survival mechanisms of microorganisms since it has been repeatedly shown that cells associated with biofilms are much less susceptible to antimicrobial agents such as antibiotics. This was first shown for bacterial species, with an increase of dosage ranging from 10- to 100-fold, depending on bacterium and antibiotic, necessary for the eradication of biofilm-associated bacteria (Donlan and Costerton 2002). Later, a similar trend was observed for fungal biofilms with drug concentrations needed for a 50 % reduction of metabolic activity being 5–8 times higher in biofilms compared to planktonic cells and minimum inhibitory concentrations (MICs) increasing 30- to 20,000-fold (Hawser and Douglas 1995). These findings were confirmed on different substrates and for different *Candida* spp. (Baillie and Douglas 1999a; Chandra et al. 2001a; Lewis et al. 2002; Ramage et al. 2001a, b).

Interestingly, Yi et al. (2011b) discovered recently that *C. albicans* biofilms formed by *MTL* (for mating type locus)-heterozygous cells differed significantly in permeability and drug resistance from their *MTL*-homozygous counterparts. The viability of cells within **a**/α biofilms was ninefold greater than that of cells in **a**/**a** and α/α biofilms after challenge of mature biofilms with 24 μg/ml fluconazole during 24–48 h. Moreover, polymorphonuclear leukocytes (PMNs) could only impregnate the upper 11 % of mature biofilms of the **a**/α-type, whereas they could penetrate the total volume of *MTL*-homozygous biofilms. The researchers propose that the *MTL*-heterozygous biofilms form the traditional, protective biofilm environment mostly found in nature and causing disease in patients, since 90 % of the free-living *C. albicans* cells are heterozygous at their mating type locus, whereas *MTL*-homozygous biofilms form a more penetrable environment which may facilitate mating (Yi et al. 2011b). Earlier, the same group had already shown that biofilms of white **a**/**a** cells were thinner than *MTL*-heterozygous biofilms, and that this could be countered by addition of a few opaque cells. In nature, it is expected that a few white cells would spontaneously switch and function as a source of pheromone of the opposite mating type, namely the product of *MFα* (Yi et al. 2011a).

Since the discovery of high drug resistance conferred by *C. albicans* biofilms, several mechanisms underlying this high antibiotic tolerance have been proposed, and these are reviewed here together with the latest advances in the field.

## Resistance to antifungals. What are the underlying reasons?

The search for safe, cheap and effective antifungals is being hindered by the great similarities between fungal cell structure and biosynthesis pathways and their mammalian counterparts. Current therapies against fungal diseases fall into five classes: (1) polyenes that bind sterols in the fungal cell membrane and cause electrolyte leakage via formation of transmembrane channels, (2) pyrimidine analogs that get incorporated in a growing RNA/DNA strand and thereby arrest fungal DNA and RNA synthesis, (3) azoles that target ergosterol biosynthesis via blockage of the enzyme lanosterol 14α-demethylase, (4) allylamines that target ergosterol biosynthesis through blocking of the enzyme squalene oxidase and (5) echinocandins that block the enzyme β-1,3-glucan synthase and thereby inhibit incorporation of β-1,3-glucans in the cell wall disturbing the integrity of the cell wall (Cowen and Steinbach 2008; Denning and Hope 2010; Ostrosky-Zeichner et al. 2010).

For the treatment of biofilms, efficacy of echinocandins and of the polyene amphotericin B lipid formulations has been shown both in vitro (Bachmann et al. 2002; Kuhn et al. 2002; Ramage et al. 2002b, 2013) and in vivo (Kucharicová et al. 2010, 2013; Mukherjee et al. 2009). The azole antifungal drugs, the pyrimidine analogs, allylamines and classic formulations of polyenes are not active against biofilms (Chandra et al. 2001a, b; Hawser and Douglas 1995; Ramage et al. 2001c). In vitro susceptibility of biofilms to antifungals is generally assessed using the 96-well microtiter plate-based method first described by Ramage et al. (2001a) (Pierce et al. 2010). Susceptibility testing under in vivo conditions is performed using catheter lock therapies, with amphotericin B, ethanol and echinocandins showing promising results, (recently reviewed by Walraven and Lee 2013) or by intraperitoneal or intravenous injection of drugs to animals that have catheter-related biofilm infections (Kucharíková et al. 2010, 2013). The underlying mechanisms possibly causing the ineffectiveness of the above-mentioned drugs are described below. To overcome the inefficacy of these drugs, more and more studies appear that focus on synergism between antifungals and antibiotics, painkillers etc., resulting in an effective combination therapy against biofilm-associated *C. albicans.* Examples of such therapies include combination of fluconazole and the tetracycline antibiotic doxycycline (Fiori and Van Dijck 2012; Gao et al. 2013), combination of amphotericin B and aspirin (Zhou et al. 2012), combination of caspofungin and the painkiller/anti-inflammation compound diclofenac (Bink et al. 2012) and the sensitization of *C. albicans* biofilms to different antifungals by the immunosuppressant drug cyclosporine a (Shinde et al. 2012).

Over the course of time a vast amount of research groups have tried to elucidate the mechanisms underlying increased resistance in biofilm-associated *C. albicans* cells. Some of the proposed causes are shared resistance mechanisms between planktonic and biofilm-associated cells (e.g. upregulation of drug efflux pumps, upregulation of target gene expression), others are biofilm specific (e.g. presence of matrix). In what follows, we highlight the major propositions and recent advances in this field (Table 1).

**Table 1:** Resistance mechanisms in *Candida albicans*

Resistance mechanism	Effect	In which growth form?
Reduced growth rate	Lower presence of antifungal targets, reducing the antifungal efficacy	Planktonic cells (Baillie and Douglas 1998)
Cell density	Quorum sensing?	Common (Perumal et al. 2007; Seneviratne et al. 2008)
Differential regulation drug targets	Changes in target levels, often associated with changes in target structure rendering the drug incapable of binding the target (White et al. 1998)	Common (Borecká-Melkusová et al. 2009; Khot et al. 2006; Nailis et al. 2010; White 1997)
Upregulation drug efflux pumps	Antifungal is pumped out of cell and can thereby not perform its intracellular function	Common (Nett et al. 2009; Ramage et al. 2002a; Sanglard et al. 1995, 1997)
Persister cells	Because of the dormant state of persisters, antifungal targets are inactive (Lewis 2010, 2012)	Biofilm (LaFleur et al. 2006)
Presence of a matrix	Specific binding of antifungals by β-1,3-glucans, a major matrix component, which prevents antifungals from reaching their targets (Nett et al. 2007b)	Biofilm (Al-Fattani and Douglas 2006; Nett et al. 2007b)
Diverse stress responses	Possibly only indirect effects via regulation of other resistance mechanisms (Robbins et al. 2011)	Common (Diez-Orejas et al. 1997; Kumamoto 2005)

Different mechanisms of resistance have been described, both for planktonic as well as for biofilm cells. In this table we indicate whether the mechanism is functional in planktonic or biofilm cells or whether the mechanism is common for both life styles

### Reduced growth rate

In general, cells that show a slow growth are more resistant. It was therefore proposed that biofilm cells are more resistant because they grow slower. However, the involvement of a reduced growth rate of biofilm cells for resistance to antifungals was renounced by Baillie and Douglas (1998). They compared amphotericin B susceptibility of biofilm-associated *C. albicans* cells with planktonic cells under different growth rates. They found that the biofilm-associated cells were resistant at all growth rates, whereas planktonic cells were only resistant when showing very slow growth (Baillie and Douglas 1998). Furthermore, Chandra et al. (2001a) showed a correlation between metabolic activity and antifungal resistance in maturing biofilms, further invalidating the effect of growth rate. Lastly, the most common used assay for quantitatively measuring biofilm formation relies on the conversion of 2,3-bis-(2-methoxy-4-nitro-5-sulfophenyl)-5-[(phenylamino)carbonyl]-2H-tetrazoliumhydroxide (XTT) to a colored formazan in the presence of metabolic activity (Kuhn et al. 2003; Paull et al. 1988). It is shown that the formazan signal corresponds very well with cell number (Hawser 1996) and generally the signal increases when a biofilm grows (Lal et al. 2010).

### Cell density

Based on the fact that the resistance of biofilms changes with (extreme) inoculum size, Perumal et al. (2007) proposed the influence of cell density on *C. albicans* drug resistance. They tested the efficacy of different azoles, amphotericin B and the echinocandin caspofungin on planktonic cells at densities similar to those found in biofilms (up to 1 × 10^8^ cells/ml) and showed that at high cell densities planktonic cells had markedly reduced susceptibilities to all drugs. These results seemed not to be associated with drug efflux or farnesol quorum sensing since a strain deficient in these mechanisms showed the same trend. Moreover, the susceptibility of dissociated biofilm cells diluted to 1 × 10^3^ cells/ml was similar to that of planktonic cells at the same cell density, indicating that the increased resistance was indeed associated with the biofilm architecture. Similar conclusions were obtained by Seneviratne et al. (2008) for the azole ketoconazole and the pyrimidine analog 5-flucytosine. However, they did not see a density-dependent susceptibility of planktonic or biofilm-associated cells to both caspofungin and amphotericin B, but they account the modified experimental procedures responsible for these discrepancies.

Therefore, cell density does seem to have an effect on *C. albicans* resistance to several drugs, but this is probably not a biofilm-specific resistance mechanism since a similar trend was observed in planktonic cells.

### Altered gene expression

Upregulation of specific genes has been shown to be involved in antifungal drug resistance in planktonic cells (Sanglard 2002; White et al. 1998). These genes can vary from genes encoding efflux pumps such as *CDR1* and *MDR1* (Sanglard et al. 1995; White 1997), which will be discussed later, to genes encoding the protein targets of antifungals such as genes involved in the ergosterol biosynthesis pathway (White 1997). The latter will cause changes in target levels, often associated with altered target structure, both resulting in the inability of the drug to effectively eradicate the pathogen (White et al. 1998). It is therefore plausible that alterations in gene expression are also responsible for drug resistance in biofilm-associated *C. albicans* cells. In this regard, expression levels of genes encoding proteins involved in the production of cell membrane and cell-wall components have been a major point of focus, with the genes involved in the ergosterol biosynthesis pathway being the most studied ones.

In a first study, mRNA levels of genes involved in ergosterol biosynthesis (the *ERG*-genes) and in β-1,6-glucan biosynthesis (*SKN1* and the *KRE*-genes) were determined via quantitative RT-PCR and compared between planktonic and biofilm-associated cells (Khot et al. 2006). The researchers found a unique transcript profile in a subpopulation of amphotericin B-resistant blastospores with a significant downregulation of *ERG1* and a significant upregulation of *ERG25*, *SKN1* and *KRE1*. Transcription levels of the latter gene also showed a correlation with increasing resistance at higher concentrations of amphotericin B. Later, the changes in *ERG*-gene expression upon addition of the azole fluconazole were investigated by Borecká-Melkusová et al. (2009) using reverse transcriptase and real-time PCR in different *C. albicans* isolates. They found upregulation of *ERG9* regardless of the susceptibility of the tested strains to fluconazole and downregulation of *ERG11* in fluconazole-susceptible strains, the product of the latter being the target of the azole class of antifungals. Detailed analysis of *ERG1* and *ERG25* expression upon addition of fluconazole showed slight increases in gene expression in both planktonic and biofilm-associated cells. A study by Nailis et al. (2010) showed a drug-specific transcription response upon challenge of biofilms with high concentrations of antifungals. They challenged biofilm-associated *C. albicans* cells with high doses of fluconazole and amphotericin B and analyzed gene expression profiles using quantitative RT-PCR. They noticed significant increases in *ERG1, ERG3, ERG11* and *ERG25* in mature biofilms upon addition of fluconazole and significant increases in *SKN1, KRE1* and *ERG1* in mature biofilms upon challenge with amphotericin B. The results of these three studies show that possible differential regulation of gene expression within biofilm-associated cells is very much depending on the experimental setup.

Recently, Yu et al. (2012) found that mature biofilms grown in the presence of farnesol, which is the precursor of ergosterol and a quorum-sensing molecule in *C. albicans*, showed a significant increase in fluconazole susceptibility compared to a farnesol-untreated biofilm. Using RT-PCR, they could show that transcription levels of *ERG1*, *ERG3*, *ERG6*, *ERG11* and *ERG25* decreased significantly in the farnesol-treated group, indicating that the ergosterol biosynthesis pathway may contribute to the inhibitory effect of farnesol and further arguing that increased transcription of the *ERG*-genes does increase biofilm resistance.

A whole transcriptome approach was applied by Vediyappan et al. (2010), who challenged mature biofilms during 2 h with fluconazole, amphotericin B and caspofungin in concentrations that were lethal for planktonic cells but not for biofilm-associated cells. Upon addition of fluconazole, only five genes were differentially expressed, causing the researchers to put forth that biofilm-associated cells might be blind to fluconazole, also explaining its inefficacy. Upon addition of amphotericin B, they saw a differential expression of 160 genes, whereas upon challenge with caspofungin the amount of differentially expressed genes increased up to a couple of hundred genes. Interestingly, this shows a correlation between antifungal susceptibility and the amount of differentially expressed genes, which is a trend opposite to what would be expected if genetic alterations are the main reason for antifungal resistance in biofilms.

An apparent contradiction rises from the studies cited here above and we want to stress that this might reflect a highly model-dependent mechanism since the in vitro model systems for biofilm formation utilized in the cited studies differed. It is for example known that the presence of medium flow during the formation of biofilms significantly alters the biofilm structure (Al-Fattani and Douglas 2006; Baillie and Douglas 2000; Hawser et al. 1998) and that the resistance to commonly used antifungals of biofilms grown under flow conditions differs significantly from statically grown biofilms (Uppuluri et al. 2009, 2011). It is therefore possible that the experimental setup for biofilm formation also influences differential gene expression in biofilm-associated cells upon challenge with antifungals, which would mean that this resistance mechanism is highly model dependent. For this reason, we do not expect altered expression of genes encoding antifungal targets to be the major resistance mechanism in biofilm-associated cells.

### Upregulation of drug efflux pumps

Upregulation of drug efflux pumps has been described as a causative factor in biofilm drug resistance for several biofilm-forming microorganisms (Soto 2013). In *C. albicans*, two groups of efflux pumps have been shown to contribute to drug resistance: the ATP binding cassette (ABC) transporters encoded by the *CDR*-genes and the major facilitator (MF) superfamily encoded by the *MDR*-genes (Ben-Yaacov et al. 1994; Fling et al. 1991; Marger and Saier 1993; Prasad et al. 1995).

An increased expression of *CDR1* (for *Candida* drug resistance) and *MDR1* (for multidrug resistance; also known as *BENr* for benomyl resistance) was first documented by Sanglard et al. (1995), in *C. albicans* clinical isolates with a high azole resistance associated with prolonged treatment. Moreover, this group showed that mutants lacking *CDR1* and *MDR1* lost their azole resistance together with resistance to other antifungals and metabolic inhibitors (Sanglard et al. 1996). Upregulation of these genes was confirmed by White (1997), and in addition they showed that members of the other families making up the ABC transporters were not involved in increased drug efflux. Later, Sanglard et al. (1997) identified a second member of the ABC transporter family, encoded by *CDR2*, which could rescue drug resistance in the highly susceptible *S. cerevisiae* multidrug transporter *pdr5Δ* mutant. Northern blotting performed on total RNA showed an increased expression of *CDR2* in resistant *C. albicans* strains. The involvement of a second member of the MF superfamily, encoded by *FLU1* (for fluconazole resistance), was discovered by usage of the same *S. cerevisiae* *pdr5Δ* mutant (Calabrese et al. 2000). So far, no other genes have been shown to be involved in this process. Efflux pump upregulation seems to primarily play a role in azole resistance (Mateus et al. 2004; Mukherjee et al. 2003; Ramage et al. 2002b; Sanglard et al. 1996) and is reported not to be involved in echinocandin resistance (Niimi et al. 2006).

An induced expression of *CDR1*, *CDR2*, *MDR1* and *FLU1* in biofilm-associated *C. albicans* cells compared to planktonic cells has been shown both in vitro (Mateus et al. 2004; Mukherjee et al. 2003; Ramage et al. 2002b) and in vivo (Andes et al. 2004; Nett et al. 2009). Increased expression of the *CDR*-genes was mainly observed after 24 h and to a lesser extent after 48 h, while *MDR1* was solely overexpressed after 24 h (Mukherjee et al. 2003; Ramage et al. 2002a). These observations already indicate that upregulation of drug efflux pumps does not play a major role in drug resistance in mature biofilms because both groups observed a decrease in efflux pump gene expression in aging biofilms whereas generally resistance increases as the biofilm ages. In fact, it seemed like challenge of the biofilm with antifungals was not necessary since adherence to a surface was enough to trigger this gene overexpression (Lepak et al. 2006; Mateus et al. 2004). Moreover, several studies have shown that *CDR1*, *CDR2* and *MDR1* single and double mutants are susceptible to azoles when grown planktonically despite retaining their resistance when grown in a biofilm structure, thereby implying that the presence of these genes is not necessary for resistance in biofilms (Mukherjee et al. 2003; Perumal et al. 2007; Ramage et al. 2002a).

Recently, it was discovered that the efflux pump encoded by *FLU1* is also responsible for efflux of the salivary human antimicrobial peptide histatin 5 (Hst5) that is toxic to *C. albicans.* Li et al. (2013) showed that *flu1Δ/Δ* had significantly reduced efflux rates of Hst5 and significantly higher cytosolic Hst5 concentrations. Moreover, this mutant showed reduced biofilm formation capacity in the presence of Hst5. RT-PCR of *C. albicans* cells showed that *FLU1* expression levels did not increase upon challenge with Hst5 in the short term, giving an indication that *FLU1*-upregulation is unlikely to become a mechanism for resistance against Hst5, showing its therapeutic potential.

In conclusion, although an increased expression of genes encoding efflux pumps has been observed in the early hours of biofilms formation, this does not seem to be the case in mature biofilms. Moreover, it has been shown that mutants lacking genes encoding efflux pumps still retain their resistance to antifungals when grown in a biofilm. These observations lead to the conclusion that upregulation of drug efflux pumps is not a major cause for increased resistance of biofilm-associated cells.

### Persister cells

Persister cells are phenotypic variants rather than mutants (Keren et al. 2004; LaFleur et al. 2006) that are able to survive antibiotic concentrations well above MICs (LaFleur et al. 2006). It is thought that the inability of an antibiotic to eradicate persister cells is a consequence of the dormant state in which persister cells are present, since antibiotics need an active target to perform their function (Lewis 2010, 2012).

Since the discovery of persister cells in 1944 (Bigger 1944), their presence has been shown in biofilms formed by different bacterial species such as *P. aeruginosa* and *Escherichia coli* (Harrison et al. 2009; Spoering and Lewis 2001) in which they make up 0.1–1 % of all cells (Keren et al. 2004). The presence of persister cells in *Candida* biofilms was first shown in 2006 when LaFleur et al. (2006) observed a biphasic killing of *C. albicans* biofilms, with the majority of the population being killed at relatively low amphotericin B concentrations and a very small fraction of cells remaining resistant even at high concentrations of the drug. 1 % of the population was completely unharmed by antifungal agents, and these cells were appointed “persisters”. Moreover, the group showed that the presence of persisters was not dependent on the formation of a complex biofilm structure, but rather on the ability to attach to a surface.

Blocking persister survival can be an interesting therapeutic option aiming at increasing *C. albicans* biofilm susceptibility to antifungals. Bink et al. (2011) discovered that superoxide dismutases, encoded by *SOD*-genes in *C. albicans* and important for detoxification of reactive oxygen species (ROS), play a major role in miconazole persistence through upregulation of *SOD*-genes upon addition of miconazole. By addition of a superoxide dismutase inhibitor *N*,*N*’-diethyldithiocarbamate (DDC) to *C. albicans* biofilms, they reduced the miconazole-resistant persister fraction 18-fold.

However, quickly after the discovery of persisters in *Candida* biofilms, it was shown that not all *Candida* strains produce persister cells (Al-Dhaheri and Douglas 2008). When the effect of AmB on biofilm formation by two *C. albicans* strains, namely SC5314 and GDH 2346, was tested, it was demonstrated that biofilms formed by the latter strain contained a small amount of cells that was resistant to AmB concentrations of 100 μg/ml after 24 h of exposure whereas the MIC for planktonic cells of this strain is 1.3 μg/ml. Surprisingly, however, biofilms formed by SC5314 showed no cells surviving after the same treatment. These results were later confirmed by a life-dead staining of biofilms cells exposed to 100 μg/ml AmB using fluorescein diacetate (Al-Dhaheri and Douglas 2010). As a consequence of these findings it can be concluded that the presence of persisters cannot be the only reason for drug resistance in *C. albicans* biofilms.

### Matrix

Cells within a *C. albicans* biofilm are embedded in an extracellular self-produced matrix (Costerton et al. 1995). The amount of matrix material present depends on the growth conditions to which the biofilm is subjected, with much more matrix material being produced when the cells are confronted with a liquid flow as compared to static conditions (Hawser et al. 1998). Like extracellular polymeric material produced by planktonic cells, the main components of the biofilm matrix are carbohydrates (glucose, mannose, rhamnose and *N*-acetylglucosamine), proteins, phosphorus, uronic acid and hexosamine (Al-Fattani and Douglas 2006; Lal et al. 2010). However, when comparing the exact composition of biofilm matrix material with its counterpart produced by planktonic cells, considerate differences were discovered concerning its carbohydrate and protein content indicating that there might be some features specific to biofilm matrix material (Al-Fattani and Douglas 2006; Baillie and Douglas 2000; Hawser et al. 1998). Recently, it was discovered that extracellular DNA (eDNA) is also an important component of biofilm matrix material, with amounts increasing over time, and that treatment with deoxyribonuclease I (DNAse) decreases biofilm biomass at later time points (Martins et al. 2010). Moreover, DNAse could enhance the activity of AmB and caspofungin on *C. albicans* cells in mature biofilms. Such a synergy was not observed with fluconazole (Martins et al. 2012).

To determine whether the presence of matrix material indeed increases the resistance of biofilms to antifungal products, Al-Fattani and Douglas (2006) grew *C. albicans* biofilms under static conditions, resulting in a small amount of matrix material, and under conditions of continuous flow using a modified Robbins device (MRD). Using scanning electron microscopy (SEM), they could confirm the presence of much more matrix material in the biofilms grown under continuous flow, compared with biofilms grown statically, as was expected. When challenging mature biofilms grown under both conditions with 5- and 30-times the MIC for planktonic cells, they found that biofilm resistance was correlated with the amount of matrix material present. In contrast, two earlier publications that compared drug susceptibility of statically grown biofilms with biofilms grown under gentle shaking did not report any differences associated with the extent of matrix formation (Baillie and Douglas 2000; Hawser et al. 1998). This might be caused by the difference in flow regimen (Al-Fattani and Douglas 2006), with the MRD, which causes a continuous unidirectional flow over the surface, possibly mimicking natural conditions more than does gentle shaking.

One potential mechanism by which matrix material increases biofilm resistance is via restricting penetration of the drug through the biofilm. This was, however, quickly confuted when Al-Fattani and Douglas (2004) showed that unless their observation that diffusion rates differed for different drugs, after 3–6 h of drug exposure, distal places in the biofilm showed drug concentrations that were several times the MICs. Even with this drug permeability into the biofilm, complete killing of biofilm-associated cells could not be accomplished.

A new light was shed on the matter when Nett et al. (2007b) discovered that cell walls of biofilm-associated cells were up to two times thicker and contained more carbohydrates and β-1,3-glucans than stationary or log-phase planktonic cells. This was true both in vitro, with supernatant of biofilms containing two to tenfold more β-1,3-glucans than supernatant of planktonic cells, and in vivo, with serum of rats with a biofilm-associated infection on a central venous catheter containing nearly tenfold more β-1,3-glucans than serum of rats with disseminated candidiasis. After isolation of matrix material, they could also show the presence of β-1,3-glucans in the biofilm matrix, and this amount was shown to increase over the course of biofilm maturation (Nett et al. 2010). Moreover, they were able to show that biofilm-associated cells could bind four to fivefold more fluconazole per cell-wall weight compared to the planktonic cells. Combining these two observations seems to indicate that β-1,3-glucans bind fluconazole in biofilm structures, thereby decreasing its potential to control biofilm-associated cells. To further support this hypothesis it was shown that both in vitro and in vivo, a combination of 1,000 μg/ml fluconazole with 1.25 units/ml zymolyase (a glucanase) could decrease biofilm viability, whereas either one separately was not able to do so (Nett et al. 2007a, b). Addition of fluconazole to biofilms at concentrations reducing metabolic activity was shown not to alter exopolysaccharide material and biofilm architecture (da Silva et al. 2012), giving an indication that binding of fluconazole to β-1,3-glucans will not affect matrix material or biofilm structure. Specific binding of antifungals by β-1,3-glucans was later shown for AmB (Vediyappan et al. 2010). Recently, Mitchell et al. (2013) showed that also in non-*albicans Candida* species, β-1,3-glucans contribute to azole resistance by specific binding.

Since this discovery, the involvement of different genes in this process has been elucidated. Firstly, the gene *FKS1*, encoding a β-1,3-glucan synthase which is the target for the echinocandin class of antifungals, was shown to be necessary for resistance, since viability of cells in a biofilm produced by a heterozygous deletion mutant which showed a 30 % reduction in β-1,3-glucans content, was reduced with 80 % after 48 h of treatment with 250 μg/ml fluconazole. A similar effect was not observed in planktonic cells (Nett et al. 2010). Furthermore, genes involved in the protein kinase C cell-wall integrity pathway, which controls cell-wall glucan content in response to stress, namely *SMI1* and *RLM1,* were shown to be essential for *C. albicans* matrix and cell-wall β-1,3-glucan content (Nett et al. 2011). Moreover, Taff et al. (2012) showed that two predicted glucan transferases, encoded by *BGL2* and *PHR1*, and one exoglucanase *XOG1*, which are predicted to be present in the extracellular matrix, are crucial for β-1,3-glucans delivery to the matrix and accumulation of β-1,3-glucans in matrix material, with biofilm-associated mutants lacking these genes showing an increased susceptibility to fluconazole. Similar phenotypes were not observed for planktonic cells. Since β-1,3-glucans are also a major component of the cell wall, the researchers propose that the three above-mentioned glucan modification proteins are also present in the cell wall. Lastly, research by Yi et al. (2011b) showed that biofilm regulation, including matrix deposition, in strains with a differential *MTL*-locus configuration involves a different pathway and different transcription factors. The more resistant *MTL*-heterozygous biofilms are regulated by the Ras1/cAMP pathway and require the subsequent action of transcription factors Efg1p, Tec1p and Bcr1p, which were termed the “transcription factor cascade”. On the other hand, the structurally similar but thinner and more permeable *MTL*-homozygous biofilms are regulated by the mitogen-activated protein kinase (MAPK) pathway and are so far only shown to require the action of transcription factor Tec1p. Most interestingly, these observations might indicate the importance of regulation of matrix deposition over general biofilm architecture in conferring antifungal resistance, but further research is needed to validate this proposition.

Whereas binding of several antifungals by β-1,3-glucans that make up a big part of *C. albicans* biofilm matrix material has been proven to reduce antifungal susceptibility of biofilm-associated cells, this cannot be the only reason for increased drug resistance in biofilms. In the first paper describing resistance of *C. albicans* biofilms (Hawser and Douglas 1995), they were grown under static conditions meaning that they contain much less matrix material than they would do in vivo where they are constantly exposed to fluid motion (Hawser et al. 1998; Hawser and Douglas 1995).

### Stress responses

During colonization of its host, *C. albicans* is confronted with a wide variety of stresses to which it responds via different conserved signal transduction pathways of which the MAPK network is a crucial component (Cannon et al. 2007; Monge et al. 2006). An important part of the MAPK network is the protein kinase C cell-wall integrity pathway that signals via the MAPK Mkc1p (Cannon et al. 2007; Navarro-Garcia et al. 1995, 1998). Whereas the importance of the cell-wall integrity pathway for virulence in a murine-disseminated *Candida* model was already published in 1997 (Diez-Orejas et al. 1997), its importance in normal biofilm formation and biofilm resistance was not known until 2005 (Kumamoto 2005) when it was demonstrated that an *mkc1*-null mutant formed an abnormal biofilm with reduced filamentation after 48 h of development. Moreover, biofilms formed by the *mkc1*-null mutant were susceptible to MICs 100-fold lower than wild-type and reintegrant strains.

Another key player in stress responses, the serine/threonine protein phosphatase calcineurin, was already known to be necessary for survival in serum and therefore for disseminated infection by *C. albicans* (Blankenship and Heitman 2005), when Uppuluri et al. (2008) showed that *Candida* strains mutated in calcineurin B (*CNB1*), which encodes the catalytic subunit of the protein, or its downstream target, the transcription factor Crz1, could be restricted by much lower fluconazole concentrations than their wild-type counterparts. In concordance with this, we have to elaborate on heat shock protein 90 (Hsp90), which interacts with the catalytic subunit of calcineurin to stabilize it and prepare it for activation (Singh et al. 2009). Hsp90 is known to be important for *C. albicans* resistance against azoles and echinocandins (Singh et al. 2009) and was shown to be necessary for biofilm dispersal and resistance to azoles in vitro and in vivo (Robbins et al. 2011). The latter might be caused by the fact that Hsp90 is a regulator of matrix glucan levels, with deletion of Hsp90 resulting in matrix material with reduced β-1,3-glucans-levels, and thus a reduced potential to capture antifungals (Robbins et al. 2011). These results indicate that a combination therapy of an Hsp90 inhibitor or calcineurin inhibitor, together with fluconazole would be an interesting therapeutic option. The potential and likelihood of *C. albicans* to develop resistance against such a combination therapy was investigated by Hill et al. (2013). They started from strains that were resistant to azoles in a manner dependent on Hsp90 and calcineurin. Of the 290 strains they started with, 7 *C. albicans* strains developed resistance to fluconazole and either geldanamycin (Hsp90 inhibitor) or FK506 (calcineurin inhibitor). Resistance mechanisms identified included: drug target mutations that conferred resistance against geldanamycin and FK506, mutations in a gene encoding a transcriptional activator of drug efflux pumps, namely *PDR1*, mutations that transformed azole resistance from dependent on calcineurin independent on this regulator and mutations in the catalytic subunit of calcineurin. Moreover, they showed extensive aneuploidy in four of the *C. albicans* lineages (Hill et al. 2013), a characteristic that has been shown to increase fitness during drug resistance development (Selmecki et al. 2009). A second heat shock protein, Hsp104, was recently shown to be important for in vitro biofilm formation and virulence in a *Caenorhabditis elegans* infection model, but a role for Hsp104 in biofilm drug resistance was not addressed in this study (Fiori et al. 2012).

From this it is clear that the high resistance to commonly used antifungals by biofilm-associated *C. albicans* cells cannot be attributed to the actions of just one mechanism, but is rather a comprehensive mechanism reflecting the complexity of the biofilm lifestyle itself.

### Escape from the immune system

The presence of pathogens in and on our bodies is generally detected by pattern recognition receptors (PRRs) that are present on different cells of our innate immune system and recognize pathogen-associated molecular patterns (PAMPs) that are either present on the cell wall of the pathogen or are secreted by the pathogen. Successful binding of a ligand by PRRs causes receptor-specific signaling through a downstream cascade, which eventually results in pathogen phagocytosis, the onset of a pro-inflammatory response via production of cytokines and chemokines and secretion of microbicidal compounds (Seider et al. 2010). The major components of the *C. albicans* cell wall, such as β-1,3-glucans, are responsible for its detection by several specific receptors, the most important ones belonging to the classes of toll-like receptors (TLRs) and C-type lectin receptors (CLRs) (Bourgeois et al. 2010; Netea et al. 2008). However, over the course of time, *C. albicans* has evolved several immune evasion strategies resulting in reduced recognition of the pathogen by our immune system. A couple of these immune evasion mechanisms include masking of specific cell-wall components to prevent PRR-mediated recognition (Galan-Diez et al. 2010; Wheeler and Fink 2006), secretion of aspartic proteases to inactivate components of the innate immune system (Gropp et al. 2009; Meiller et al. 2009), switching to the opaque form with reduced filamentation to circumvent recognition mechanisms based on the hyphal state (Sasse et al. 2013) and expression of surface proteins such as Pra1p and Gpd2p that actively bind factor H and FHL1 thereby mimicking host cells resulting in protection from the complement system (Luo et al. 2009; Luo et al. 2013).

Analysis of the interaction between biofilm-associated *C. albicans* cells and our immune system started only recently but has already shown to be very distinct from interactions with planktonic *C. albicans* cells. In their research, Chandra et al. (2007) showed that peripheral blood mononuclear cells (PBMCs) did not phagocytose biofilm-associated cells, as opposed to planktonic cells. In contrast, the presence of PBMCs during biofilm development enhanced the process with significantly thicker biofilms being formed as a consequence of unknown factors secreted by the immune cells. Comparable to this, it was found that the presence of the pro-inflammatory cytokine IL-17A enhanced *C. albicans* biofilm formation in vitro (Zelante et al. 2012). These data support the idea that biofilm formation might be an adaptation to survival within the hostile environment inside the host.

By a mechanism that is still unknown, biofilm-associated *C. albicans* cells can change the profile of cytokines secreted by PBMCs (Chandra et al. 2007) and phagocytes (Katragkou et al. 2010). Furthermore, when biofilms were exposed to the echinocandin anidulafungin, the cytokine profile secreted by the phagocytes was altered once more toward a more beneficial Th1 response (Katragkou et al. 2010) thereby steering our immune system into eradication of the invasive fungal infection (Kullberg et al. 2004).

Infiltration of immune cells into the biofilm structure has been reported repeatedly. In in vitro studies, PBMCs and PMNs were shown only to be present in the top and middle layers of most biofilms (Chandra et al. 2007; Yi et al. 2011b), whereas the less frequently encountered, more penetrable *MTL*-homozygous biofilms possessed PMNs distributed over their whole volume (Yi et al. 2011b). In an in vivo mouse model of oropharyngeal candidiasis, clusters of neutrophils were found to be present in the mucosal biofilm structure (Dongari-Bagtzoglou et al. 2009).

Reduced activity of innate immune cells on biofilm-associated *C. albicans* cells was also shown by Katragkou et al. (2010). They demonstrated that the potential of phagocytes to kill *C. albicans* was reduced for biofilm-associated cells, compared to planktonic cells and resuspended biofilm cells, similar to the above-mentioned behavior of PBMCs on biofilm-associated cells discovered by Chandra et al. (2007). Exposing the biofilms to sub-inhibitory concentrations of anidulafungin (0.12 mg/l) led to a significant increase in phagocyte induced damage, which, according to them, might be caused by an increased exposure of β-1,3-glucans which are important PAMPs. The hypothesis that cells are primarily protected by mature biofilms was established by Xie et al. (2012). When 3-h old biofilms were exposed to HL-60 (a human neutrophil-like cell line) cells they lost over 80 % of their activity, whereas the activity of 24- and 48-h biofilms was only reduced with less than 30 %. Consistent with this, mature biofilms did not elicit a robust oxidative response, which is one of the main mechanisms by which neutrophils kill pathogens, in sharp contrast with 3-h old biofilms. Moreover, dispersed 24-h biofilm cells also failed to prevent a ROS response, leading the group to suspect a role for the biofilm matrix. This role was confirmed when biofilm matrix alone did not trigger a reactive oxygen response, and the true protector was unmasked when glucanase treatment of the matrix completely abrogated the matrix ROS-attenuating effect.

## Conclusion

Reducing the incidence of biofilm-related candidemias in hospitals is a requirement in the search for optimized patient care. However, the high degree of resistance of biofilm-associated *C. albicans* cells hinders rapid development toward highly efficacious therapies. Recent efforts of various excellent research groups tremendously broadened our knowledge on the complex mechanisms underlying biofilm resistance. According to the authors, the presence of matrix material is the most important biofilm-resistance mechanism. Its involvement has been shown by several elegant experiments and the fact that it is only present in biofilms can explain the increased susceptibility of planktonic cells and resuspended biofilm cells. However, we do expect that several less important mechanisms such as cell density, differential regulation of drug targets, upregulation of drug efflux pumps in developing biofilms, the presence of persisters in biofilms, upregulation of different pathways associated with stress responses and possibly yet undefined mechanisms can further increase resistance to a maximum level. The elucidation of these resistance mechanisms provides a promising step toward the development of optimal therapies. Such therapies can include classic antifungal therapies including catheter lock therapies, combination therapies, natural compounds (Sardi et al. 2013) and immunotherapies that are gaining more and more attention. To enable us to develop the full potential of immunotherapies, lot of effort is being put in revealing the specific interaction of biofilm-associated *C. albicans* cells with components of our immune system.
